# Analysis of C-reactive protein from finger stick dried blood spot to predict high risk of cardiovascular disease

**DOI:** 10.1038/s41598-023-27522-6

**Published:** 2023-02-13

**Authors:** Michael Y. Schakelaar, Hans Kemperman, Arjan H. Schoneveld, Imo E. Hoefer, Wouter M. Tiel Groenestege

**Affiliations:** Central Diagnostic Laboratory, University Medical Center Utrecht, Heidelberglaan 100, 3584 CX Utrecht, The Netherlands

**Keywords:** Cardiovascular biology, Risk factors

## Abstract

C-reactive protein (CRP) is an acute-phase protein involved in inflammation. Furthermore, CRP is an important biomarker used in diagnostics to predict risk of cardiovascular disease (CVD) in addition to monitoring bacterial and viral infections. To measure plasma CRP, venipuncture is still necessitated and has to be performed by trained phlebotomists. As a solution, dried blood spots (DBS) are used for minimally invasive at-home sampling of blood and can be send to diagnostic laboratories by regular mail. In this study, we included 53 patients that presented to the outpatient clinic of the University Medical Center Utrecht. Capillary finger stick was used to spot blood on a filter paper card and allowed to dry. After extraction of DBS, CRP was analyzed on an automated high-throughput chemistry analyzer. Additional validation steps regarding stability, effect of hematocrit, precision, and limits of blank and quantitation were conducted according to corresponding Clinical and Laboratory Standards Institute standards. An excellent regression analysis of R^2^ (95% confidence interval) = 0.986 (0.982–0.989) was found. This enabled correct classification for high CVD risk of all 25 cases with sensitivity (95% CI) of 1.00 (1.00–1.00) and specificity (95% CI) of 0.96 (0.89–1.03) and correct diagnosis of inflammation of 12/13 cases with sensitivity (95% CI) of 0.92 (0.77–1.07) and specificity (95% CI) of 1.00 (1.00–1.00). Furthermore, CRP was found to be stable for 31 days and observed hematocrit variation amongst patients was clinically acceptable. CRP from DBS can be accurately measured on an automated high-throughput chemistry analyzer and used to diagnose inflammation and classify high CVD risk. This method enables individuals to engage in at-home sampling of blood on DBS for (tele)diagnostics, screening programs, patient follow-up, and medication management.

## Introduction

C-reactive protein (CRP) is synthesized by the liver as an acute-phase protein and is involved in inflammation. As a component of the innate immune system, it is known to be protective against bacterial and viral infections and may aid in the development of cardiovascular disease (CVD)^1^. In hospitalized patients with coronavirus disease 2019 (COVID-19), high CRP readouts were linked to acute kidney injury, venous thrombo-embolism, critical illness, and death when compared to low CRP levels^2^.

In the blood vessel, CRP is involved in endothelial dysfunction that consequently leads to atherosclerotic plaque development and progression^3^. Multiple studies^4–7^ already confirmed a correlation between increased CRP levels and (long-term) cardiovascular health. Therefore, incorporating CRP into CVD risk models improves the prediction of atherosclerosis and myocardial infarction^8,9^. CRP levels can be classified as low, intermediate, and high risk of CVD within one year (CRP < 1.0, 1.0–3.0, > 3.0 mg/L respectively)^10,11^. Beside CVD risk assessment, CRP is used for diagnosis of possible infections, with generally, a cut-off of CRP ≥ 10 mg/L for inflammation positive diagnosis^12^.

Because CRP is of prognostic value in many diseases, it makes an interesting target as a biomarker in screening or patient follow-up. To measure plasma CRP, venipuncture is still necessitated and has to be performed by trained phlebotomists. For patients, this requires traveling to a designated sampling locations, often a medical clinic or hospital, and a relatively painful sampling experience^13^. Alternatively, dried blood spots (DBS) may offer a solution for single CRP measurements. With a small lancet, regularly used by diabetics, capillary blood sampling can be done at home without the need for medical personnel. CRP has first been analyzed from DBS in 1991 in patients with cystic fibrosis^14^. Since then, several studies^15,16^ have concluded that CRP is accurately detectable from DBS and other groups^17–20^ have implemented this in diverse research fields on small scale. However, to date, there is no evidence that CRP from DBS can be used in everyday diagnostic practice. This would require high throughput and automated analysis of DBS cards instead of labor-intensive and small-scale non-automated immunoassays.

In the present study, we investigated if DBS-derived CRP measurements can be implemented in routine diagnostics. To this end, we performed a method comparison between venous plasma and finger stick DBS extracts with an excellent correlation of R^2^ = 0.99 which enabled correct diagnosis of inflammation and correct classification for high CVD risk assessment. All experiments were run on a completely automated high-throughput Atellica CH Analyzer, therefore our findings can be quickly translated to the clinic where it enables (tele)diagnostics, large-scale screening programs, patient follow-up, and medicine management in a patient friendly manner.

## Materials and methods

### Participants

Venous DBS method comparison whole blood samples were collected from anonymized leftover patient samples that presented to the Central Diagnostic Laboratory of the UMC Utrecht (Utrecht, The Netherlands).

For the finger stick DBS method comparison, blood samples were collected from patients, presenting to the outpatient clinic of the UMC Utrecht for CRP analysis, after giving written informed consent. An overview of both study population characteristics is presented in Table 1. The number of subjects included complies with Clinical and Laboratory Standards Institute (CLSI) evaluation protocol EP09c. The study was performed under the tenets of the Helsinki Declaration (as revised in 2013) and all relevant national regulations and institutional policies. The study protocol was approved by the authors’ Institutional Review Board (Medisch-Etische Toetsingscommissie (METC) NedMec 20–676/C).

**Table 1:** Study population overview.

	Method comparison of plasma CRP with CRP from venous whole blood DBS	Method comparison of plasma CRP with CRP from finger stick DBS
**CRP (mg/L)**		
Mean	29	9
Range	1–146	1–87
**Age (years)**		
Mean	57	58
Range	1–87	18–85
**Sex**		
Male (n)	50	32
Female (n)	50	21

### Sample collection

Venous blood sampling was performed via venipuncture with the BD Vacutainer^®^ blood collection system (Becton Dickinson, NJ, USA), at the cubital fossa and collected in either K_2_EDTA tubes (K_2_EDTA 2 mL 3.6 mg or K_2_EDTA 4 mL 7.2 mg EDTA, BD Vacutainer^®^, Plymouth, UK) or Lithium Heparin tubes (Lithium Heparin 3 mL 56 U USP or Lithium Heparin 10 mL 158 U USP, BD Vacutainer^®^, Plymouth, UK). In addition, part of venous samples was spotted on filter paper to examine correlation between venous plasma and venous DBS CRP. Therefore, 40 µl of venous EDTA whole blood was spotted on CF12 grade Whatman filter paper which meets CLSI quality criteria for e.g. pH, mean serum uptake, mean blood absorption time, and mean spot diameter (ISO9001 certified, GE Healthcare Life Sciences, Buckinghamshire, UK) and left to dry at room temperature for at least 24 h unless indicated otherwise.

In addition to the venipuncture, capillary blood samples were obtained at the same moment by the finger stick technique. If necessary, the skin site was warmed before the puncture with a gel pack of 42 °C for maximal 3 min. First, the puncture site was cleaned with a tissue containing isopropyl alcohol 70% and allowed to dry. The skin was punctured using a BD Microtainer^®^ contact activated lancet (Becton Dickinson, NJ, USA) to a depth of 2.0 mm and the first drop of blood was wiped off. The finger was gently intermittently squeezed to produce blood drops, which were collected on Whatman CF12 grade filter paper (ISO9001 certified, GE Healthcare Life Sciences, Buckinghamshire, UK). After collection, filter cards were left to dry at room temperature for at least 24 h.

### DBS extraction optimization for CRP

**Figure 1:**
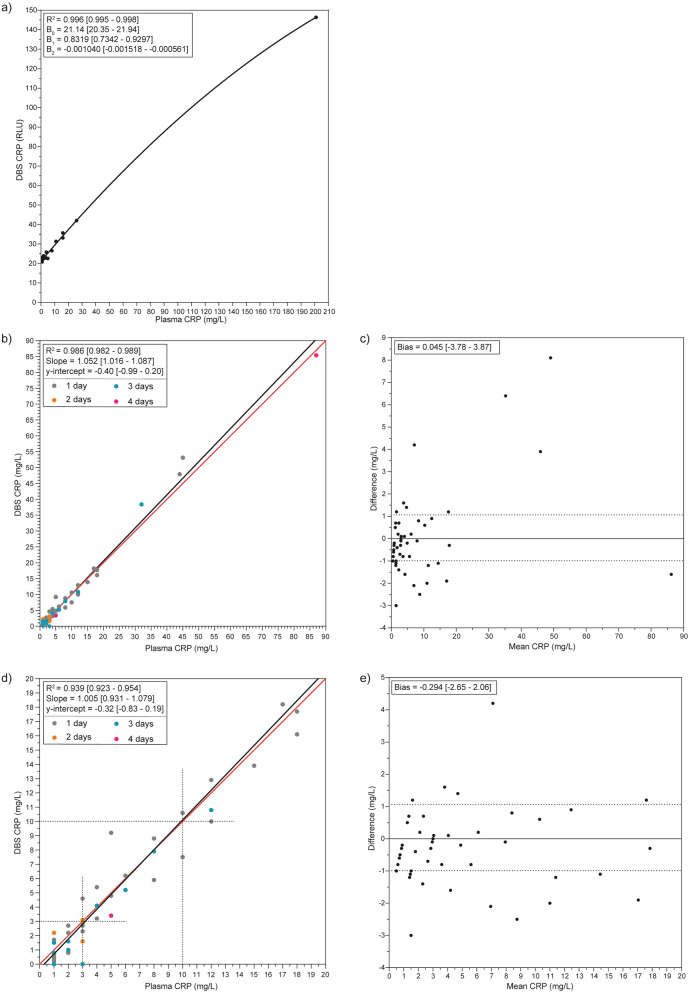
CRP method comparison. (**A**) Calibration line for DBS CRP, calibration line was calculated with second-order polynomial regression; n = 19. (**B**) Method comparison for DBS CRP with the line of identity (red) and Deming linear regression (black). Storage time of DBS cards is displayed in grey (1 day, n = 34), orange (2 days, n = 4), blue (3 days, n = 13), and pink (4 days, n = 2); total n = 53. (**C**) Bias plot from method comparison of panel (**B**). (**D**) Method comparison for DBS CRP in the near normal range with the line of identity (red), Deming linear regression (black), and cut-off values for diagnosis (black dotted lines). Storage time of DBS cards is displayed in grey (1 day, n = 32), orange (2 days, n = 4), blue (3 days, n = 16), and pink (4 days, n = 1); total n = 49. (**E**) Bias plot from method comparison of panel (**D**). All 95% confidence intervals are shown in between brackets.

**Figure 2:**
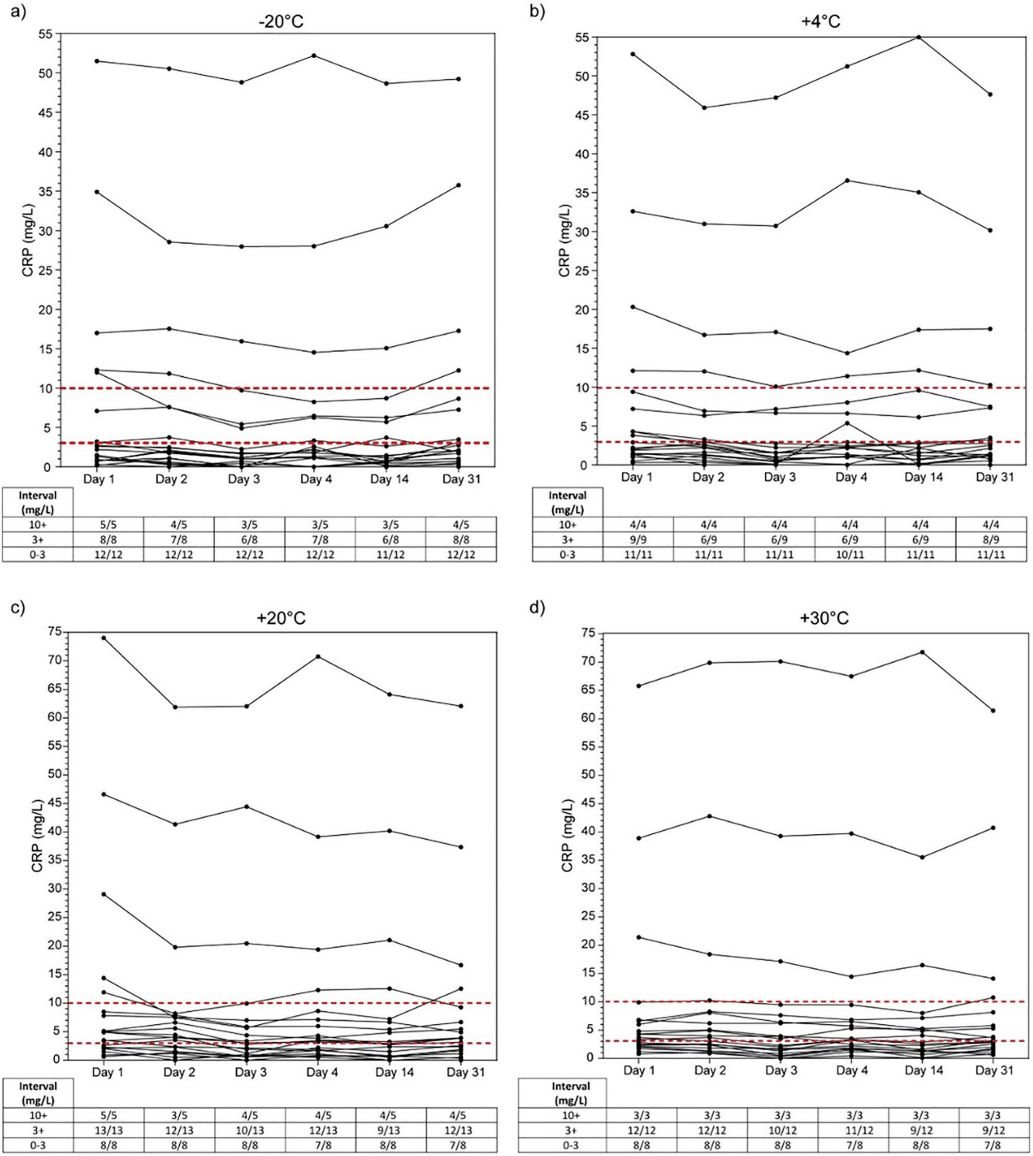
CRP stability. Stability of 20 patient samples after storage up to 31 days at temperatures of (A) -20 °C, (B) + 4 °C, (C) + 20 °C, and (D) + 30 °C. Clinically relevant cut-off values are represented by red lines in each panel. Under each panel, a table with a classification of patient samples per CRP interval is visible (X/X = number of samples at day X/number of samples at day 1).

After drying, 3 × 8 mm punches were transferred to 2 ml Eppendorf cups (Eppendorf AG, Hamburg, Germany) and 150 µl PBS (pH 7.4, Gibco, Life Technologies Europe B.V., Bleiswijk, The Netherlands) was added per spot. Samples were incubated at room temperature for 15 min on an orbital shaker (VWR Microplate Shaker, Haasrode, Belgium) at 600 rpm. Thereafter, samples were centrifuged at 21130×*g* for 3 min at room temperature and supernatant was transferred to analyzer cups (Tube-Top sample cups, Atellica^®^ Solution, Siemens Healthcare Diagnostics Inc., Tarrytown, USA). CRP was measured on an Atellica CH Analyzer (Siemens Healthcare Diagnostics Inc., Tarrytown, USA) according to the manufacturer’s instructions. The results of various extraction protocols tested and of proportional relation between number of DBS and extraction buffer volume are shown in Supplementary (Sup.) Figs. 1, 2.

### Calibration, quality control, and method comparison

To convert CRP concentration from DBS to plasma-equivalent concentrations, two calibration lines were prepared by spotting either venous EDTA whole blood samples (K2E Coagulation 3.6 mg EDTA, 2 or 4 ml, BD Vacutainer^®^, Plymouth, UK) or capillary finger stick whole blood samples that were selected based on CRP levels measured in heparin plasma used for routine diagnostics (BD Vacutainer^®^ Barricor LH plasma tube, Becton Dickinson, NJ, USA) which were sampled at the same blood draw. Whole blood of 28 and 19 patients was spotted, extracted, and measured as described above for venous and capillary calibration lines, respectively. Venous calibration line was used for method comparison (Sup. Fig. 3) validation of stability, hematocrit effect, precision, and limit of blank and quantitation. Study populations for the method comparisons of CRP from venous EDTA whole blood DBS versus venous heparin plasma and for CRP from finger stick whole blood DBS extracts versus venous heparin plasma are described in Table 1. Samples were spotted, extracted, and analyzed as described above.

To check calibration line quality, established QC samples (Liquid Assayed Multiqual Premium Levels 1 (3.8 mg/L), 2 (28 mg/L), and 3 (60 mg/L), Bio-Rad Laboratories, Clinical Diagnostics Group, California, USA) were spotted, extracted, and measured 20-fold over three days to calculate mean and standard deviation (SD). During each method comparison experiment, calibration line was checked through measuring QC samples occasionally and excluding batches from analysis that exceeded ± 2 SD or limits of acceptance ± 0.5 × CV_i_ in which CV_i_ describes the coefficient of variation within a subject obtained from literature^21^.

### Limit of blank, limit of detection, and limit of quantitation

The Limit of Blank (LoB) and Limit of Quantitation (LoQ) were determined based on CLSI protocol EP17-A2. Limit of Detection was calculated according to literature^22^. For LoB, 2 venous EDTA whole blood blank samples (K2E Coagulation 3.6 mg EDTA, 2 or 4 ml, BD Vacutainer^®^, Plymouth, UK) which demonstrated a plasma CRP concentration below the detection limit on Atellica CH Analyzer (Siemens Healthcare Diagnostics Inc., Tarrytown, USA) were selected. Each blank sample was spotted, extracted, and analyzed in ten replicates. For LoQ, six samples with different low CRP concentrations were spotted, extracted, and measured in duplicates. For both, the protocol was repeated for three consecutive days and with two different reagent LOTs to obtain a total of 60 and 36 replicates per reagent LOT for LoB and LoQ, respectively.

### Precision

Precision of CRP was determined in accordance with CLSI protocol EP15-A3. EDTA whole blood patient pools (n = 3) with matching blood types were made with target concentrations of 1.5 mg/L, 9.5 mg/L, and 106 mg/L. For five consecutive days, every pool was spotted and extracted four times per day and measured in duplicate.

### Time and temperature stability of CRP from DBS

EDTA whole blood of 20 patients was spotted and allowed to dry for 3 h at room temperature before storing under varying conditions. Cards were stored in zip-lock plastic bags at temperatures of − 20 °C, + 4 °C, 20 °C, and + 30 °C for 1, 2, 3, 4, 14, and 31 days. After appropriate storage time, DBS were analyzed according to protocol in one run.

### Effect of hematocrit on CRP concentration from DBS

EDTA whole blood patient pools (n = 5) with matching blood types with target concentrations of 3.6 mg/L, 8.6 mg/L, and 50 mg/L were made. Then, pools were centrifuged at 2500×*g* for 7 min at room temperature to separate blood cells from plasma. The plasma fraction was removed and aliquoted to obtain samples with the same CRP concentration. Thereafter, the cell fraction of the centrifuged pool was added to create a hematocrit (Hct) range. After mixing samples by inversion, Hct was analyzed by a Cell-Dyn Sapphire Hematology Analyzer (Abbott, Abbott Park Illinois, USA). Then, all samples were spotted, extracted, analyzed, and plotted against measured Hct values.

### Statistical analysis

The calibration line was generated using linear regression analysis in GraphPad Prism 9 (Version 9.3.0 (463), GraphPad Software, LLC, San Diego, USA). Method comparison data were analyzed using EP Evaluator^®^ (Data Innovations, VT, USA, version 12.2). 95% CIs for R^2^ data was calculated according to R^2^ ± 2 × SE_R2_ where ${SE}_{R2}=\sqrt{\frac{{4R}^{2}(1-{R}^{2}{)}^{2}(n-k-1{)}^{2}}{{(n}^{2}-1)(n+3)}}$ in which k is the number of independent variables and n is the number of cases.

Diagnostic sensitivity and specificity for both high CVD risk and inflammation were calculated using the following formula’s: $sensitivity = \frac{true \,positive}{{true\, positive + false\, negative}}$ and $specificity = \frac{true \,negative}{{true\, negative + false\, positive}}$. In addition, we reported 95% confidence intervals (CI) for sensitivity and specificity as follows: $95\% CI = \left[ {sensitivity\, or\, specificity} \right] \pm 1.96*SE_{{\left[ {sensitivity \,or\, specificity} \right]}}$ where $SE_{{\left[ {sensitivity\, or \,specificity} \right]}} = \sqrt {\frac{{\left[ {sensitivity\, or\, specificity} \right] - \left( {1 - \left[ {sensitivity \,or \,specificity} \right]} \right)}}{{n_{{\left[ {sensitivity \,or\, specificity} \right]}} }}}$.

LoB, LoQ, and precision data were calculated and analyzed using EP Evaluator^®^ (Data Innovations, VT, USA, version 12.2) following software instructions.

Statistical analysis for the hematocrit experiment was performed in GraphPad Prism 9 (Version 9.3.0 (463), GraphPad Software, LLC, San Diego, USA) by creating a linear regression line. With the linear regression line, an absolute and percentage of CRP decrease per 1% increase in Hct was calculated for a baseline Hct of 45%.

## Results

### Precision, limit of blank, limit of detection, and limit of quantitation

Precision results are shown in Table 2. At low CRP concentration, within run and between run variation is large but when CRP concentration increases the variation decreases rapidly. LoB, LoD, and LoQ were established at 0.55 mg/L, 1.87 mg/L, and 3.7 mg/L, respectively.

**Table 2:** DBS CRP precision.

Mean concentration (mg/L)	Within run CV (%)	Between run CV (%)	Between day CV (%)	Total CV (%)
1.5	43.7	31.0	0.0	53.6
9.5	4.5	7.1	6.0	10.4
106	1.1	2.5	3.5	4.4

### Validation of DBS-derived CRP measurements on a Siemens Atellica CH high-throughput chemistry analyzer

To convert CRP concentration from DBS to plasma-equivalent concentrations, a calibration line with R^2^ (95% CI) = 0.996 (0.995–0.998) (Fig. 1A) was used in all experiments with finger stick DBS samples.

For the method comparison of venous heparin plasma CRP versus finger stick DBS CRP, 53 patients were analyzed. Cards were dried and stored for minimally 1 and maximally 4 days after validating CRP stability (Fig. 2). It was shown that R^2^ (95% CI) = 0.986 (0.982–0.989), slope (95% CI) = 1.052 (1.016–1.087), and y-intercept (95% CI) = − 0.40 (− 0.99 to 0.20) (Fig. 1B) with a bias (95% CI) of 0.045 (− 3.78 to 3.87) (Fig. 1C). When excluding the CRP measurements > 20 mg/L, we observed R^2^ (95% CI) = 0.939 (0.923–0.954), slope (95% CI) = 1.005 (0.931–1.079), and y-intercept (95% CI) = − 0.32 (− 0.83 to 0.19) (Fig. 1D) with a bias (95%) of − 0.294 (− 2.65 to 2.06) (Fig. 1E).

When using a previously determined cut-off of CRP > 3 mg/L which corresponds to high risk for developing cardiovascular disease^10,11^ it is possible to classify the studied population accordingly. Comparing all samples that had plasma CRP > 3 mg/L with classification based on DBS gave a sensitivity (95% CI) of 1.00 (1.00–1.00) and specificity (95% CI) of 0.96 (0.89–1.03).

Additionally, CRP ≥ 10 mg/L indicates the presence of inflammation^12^. After classification of the population based on DBS read-outs, a sensitivity (95% CI) of 0.92 (0.77–1.07) and specificity (95% CI) of 1.00 (1.00–1.00) was observed.

### CRP stability up to 31 days at temperatures ranging from − 20 °C to + 30 °C

To investigate CRP stability, all samples were separated in three categories of clinical relevance (group 1 = 10 + mg/L, group 2 = 3 + mg/L, and group 3 = 0–3 mg/L) according to literature^10–12^. In most cases, patient samples remained in the category that was determined on day 1 (Fig. 2). Thus, clinical decision-making is not influenced significantly by storage of CRP in DBS for up to a month between− 20 and + 30 °C.

### Hematocrit effect on DBS CRP measurement

**Figure 3:**
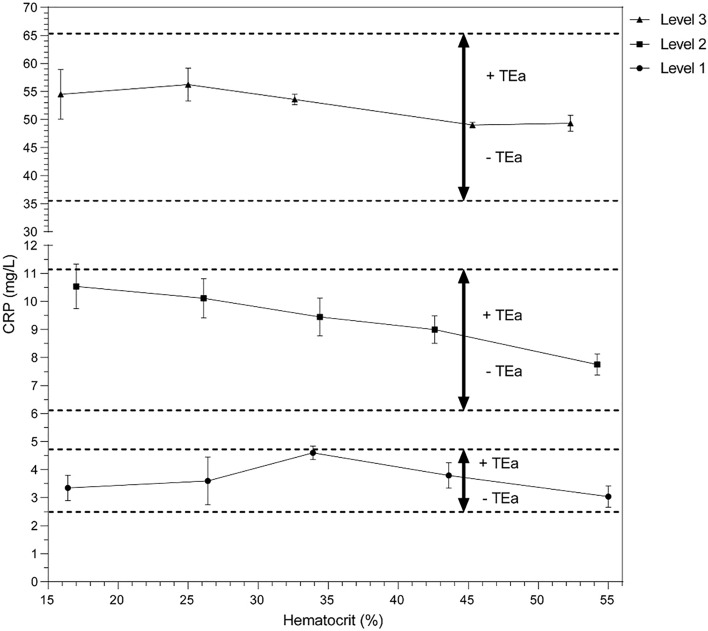
Effect of hematocrit on CRP. Pools from five patients with three different target concentrations were made. From each pool, a hematocrit dilution range was created and real hematocrit was measured with a Cell-Dyn Sapphire Hematology Analyzer (Abbott, Abbott Park Illinois, USA). Then, blood was spotted on DBS cards and stored for 1 day at room temperature before analysis. From a normal hematocrit of 45%, total allowable error (TEa) intervals were plotted with a dotted line.

If a metabolite, like CRP, is only present in the plasma fraction of blood, hematocrit variation amongst patients may induce measurement errors due to the inability to separate plasma and cytoplasm of blood cells in DBS^23^. To this end, patient pools with different target concentrations (3.6, 8.6, and 50 mg/L) were tested with a Hct range of 16–55% (Fig. 3). At the highest CRP concentration of 50 mg/L, CRP concentration after extraction decreased by 0.19 mg/L for every percent increase in hematocrit. Also at lower CRP concentrations this effect is observed (Table 3). Despite this effect, CRP concentration remains well within optimal TEa limits of ± 25.4% as described in literature^21^ along the entire Hct range examined (Fig. 3).

**Table 3:** Effect of hematocrit on CRP. From the data in Fig. 3, a simple linear regression analysis provided the slope and 95% confidence interval (CI), Hct = hematocrit.

CRP concentration (mg/L) at Hct = 45%	CRP decrease (mg/L) per 1% Hct increase	95% CI (mg/L)	CRP decrease (%) per 1% Hct increase	95% CI (%)
3.6	0.007	− 0.002 to 0.004	0.19	− 0.05 to 0.11
8.6	0.07	0.05–0.10	0.81	0.58–1.16
50	0.19	0.08–0.30	0.38	0.19–0.59

## Discussion

In the present study, we describe a simple and fast extraction protocol for CRP measurements from DBS that can be performed with common laboratory instruments and reagents (Sup. Figs. 1, 2). DBS-derived CRP can be measured on an automated high-throughput chemistry analyzer with routine CRP assay and reference intervals whilst maintaining excellent accuracy (Fig. 1). Furthermore, CRP from DBS remains stable up to 31 days under varying temperatures (Fig. 2). The effect of hematocrit variation is limited and does not result in clinically relevant variation (Fig. 3). Our data propose the use of DBS for CRP measurements in existing routine diagnostics so that (tele)diagnostics, (international) screening programs, risk analyses, and patient follow-up can be made easier and minimally invasive for both patients and laboratories.

To use DBS for CRP diagnostics, great measuring precision at low CRP concentration is crucial in delivering reliable results. Table 2 shows excellent precision data for CRP from DBS and when compared to the assay manual for CRP on the Atellica CH Analyzer^24^, a comparable or better precision is found at 9 + mg/L concentrations. On the contrary, at very low CRP concentrations, DBS precision is too low to give accurate results. LoB, LoD, and LoQ data support these findings. Also, the number of DBS used in extraction is proportionally related to the buffer volume (Sup. Fig. 2) which allows for further upscaling if more tests are validated for DBS usage.

In this study, high CVD risk assessment was investigated by classification of the population with CRP > 3 mg/L according to the literature^10,11^. All 25 patients with plasma CRP > 3 mg/L were confirmed with DBS CRP analysis and only one false positive was observed. Furthermore, the presence of infection was checked according to literature cut-offs^12^. 12/13 patients with heparin plasma levels of CRP ≥ 10 mg/L were confirmed after DBS analysis, without giving any false positives. The only patient that was misdiagnosed had a DBS CRP of 10 mg/L, exactly on the cut-off value. Consequently, we can conclude that DBS-derived CRP can be used to assess high CVD risk and diagnose state of inflammation accurately and reliably. This method can be used to classify patients that are at high risk of developing CVD however, this method does not enable to classify patients with low and intermediate risk of CVD, and therefore, cannot be considered as a high sensitive CRP assay.

The stability of CRP from DBS has been described most recently in 2005 when it was concluded that CRP recovery is 110% after 1 month at − 24 °C and that CRP from DBS can be used in long-term biobank storage^25^. Here, worse lower range CRP (0–3 mg/L) stability is likely due to the larger CV in this range (Table 2). However, correct high CVD risk assessment and inflammation diagnosis is still possible, thus enabling transport of DBS cards without the need for temperature regulation in most countries. Also, prolonged stability is important for sampling in areas with underdeveloped infrastructure where it takes longer than a few days from sampling to analysis. However, to certify CRP stability in DBS, further experiments with varying humidity are required. One limitation of measuring CRP form DBS is the longer turnaround time compared to plasma analysis. Therefore, DBS is not the method of choice for detection of acute inflammation requiring immediate action. However, in addition to assess high CVD risk, measurement of CRP from DBS could have clinically added value in the follow-up of *e.g.* rheumatoid disease, Crohn’s disease, and sarcoidosis, from the comfort of the patients home.

Another limitation of DBS is the inability to separate blood cells from plasma. Therefore, analysis of metabolites that are only present in plasma might be affected by interpatient Hct variations^23,26^. Along a wide range of Hct (from 16 to 55%), DBS-derived CRP was found to be minimally affected by Hct and remained well within the optimal TEa. For that reason, we concluded that Hct does not influence CRP in a way relevant to diagnostic practice. Furthermore, the amount of CRP decrease per percent Hct increase is dependent on CRP concentration, as higher CRP levels result in a larger decrease (Table 3). In an attempt to correct for any Hct effect in our method comparison (Fig. 1), Hct, hemolysis-index, lactate dehydrogenase, and lithium, in pre-spotted lithium chloride cards were used as a correction factor. Surprisingly, these analyses did not produce better correlation data (data not shown).

## Conclusions

CRP and other metabolites from DBS have already been used in several clinical studies^27,28^ but have never been implemented in existing routine diagnostics. All our data is obtained via analysis on the automated high-throughput Atellica CH Analyzer, a routine diagnostics analyzer. Therefore, existing reagents and reference intervals can be used for this application, allowing quick upscaling. If implemented, patients save traveling time and costs as they can perform at-home sampling and laboratories can save on sample transport and phlebotomists costs. The data are promising to use the DBS method in a routine setting. To determine clinical sensitivity and specificity, the next step will be to test this DBS method in practice with a larger patient cohort, including the self-sampling at home and monitoring possible additional problems. In the future, more analytes can be validated for DBS measurements so that more elaborate test panels can be introduced.

## Supplementary Information

Supplementary Information.

## Data Availability

The raw data supporting the conclusions of this article will be made available by the corresponding author upon reasonable request.
