# Supplementary material for: Analysis of C-reactive protein from finger stick dried blood spot to predict high risk of cardiovascular disease
Source: Sci Rep. 2023 Feb 13;13:2515. doi: 10.1038/s41598-023-27522-6 (PMC9923659; doi:10.1038/s41598-023-27522-6)
Supplement: Supplementary file 1 — Supplementary Information. [file 41598_2023_27522_MOESM1_ESM.pdf]

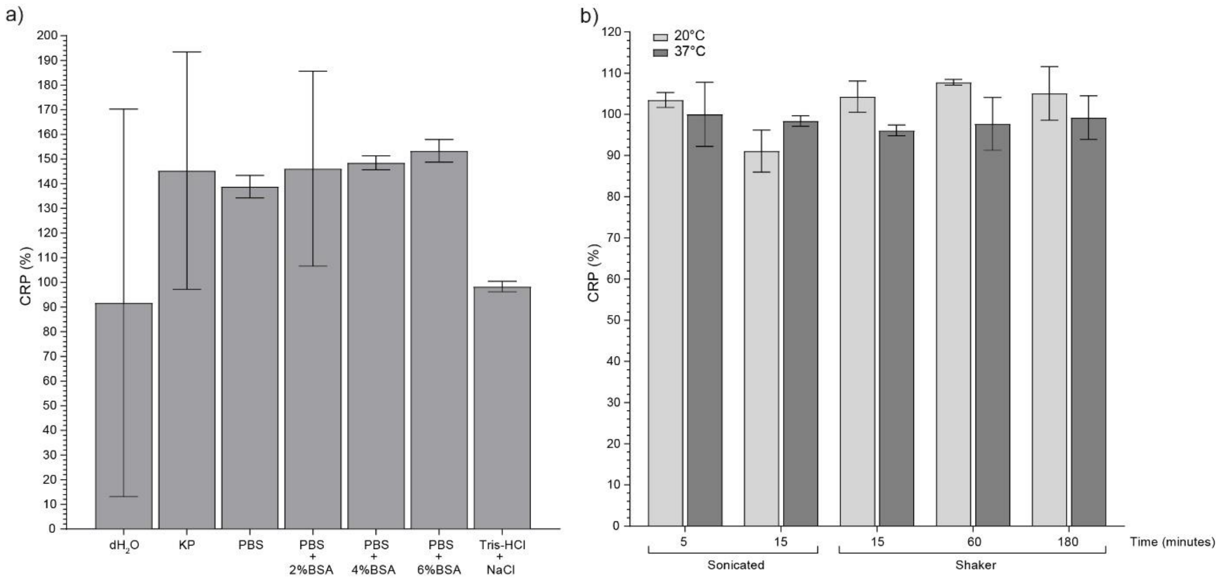

Supplementary figure 1 – Extraction optimization.

(A) Whole blood was spotted on DBS cards and stored for 1 day before extraction. 8 mm punches were collected in 2 ml Eppendorf tubes and 300 µl extraction buffer (distilled water, potassium phosphate buffer (0.698 M KH_2PO_4 + 0.3014 M K_2HPO_4, pH = 6.5), PBS (pH 7.4, Gibco, Life Technologies Europe B.V., Bleiswijk, The Netherlands) + BSA (Sigma chemical co., St. Louis, USA), and Tris-HCl/NaCl (40 mM Tris-HCl, 100 mM NaCl, pH = 7.4) was added. Samples were incubated on a shaker for 3 hours at room temperature before centrifugation at 21,130 × g for 3 minutes. Then, the supernatant was analyzed for CRP. (B) Whole blood was spotted on DBS cards and stored for 1 day before extraction. 8 mm punches were collected in 2 ml Eppendorf tubes and 300 µl PBS (pH 7.4, Gibco, Life Technologies Europe B.V., Bleiswijk, The Netherlands) was added. Samples incubated on a sonicator or shaker for different amounts of time at different temperatures before the supernatant was analyzed for TSH, dH_2O = distilled water, KP = potassium phosphate buffer, PBS = phosphate-buffered saline, BSA = bovine serum albumin.

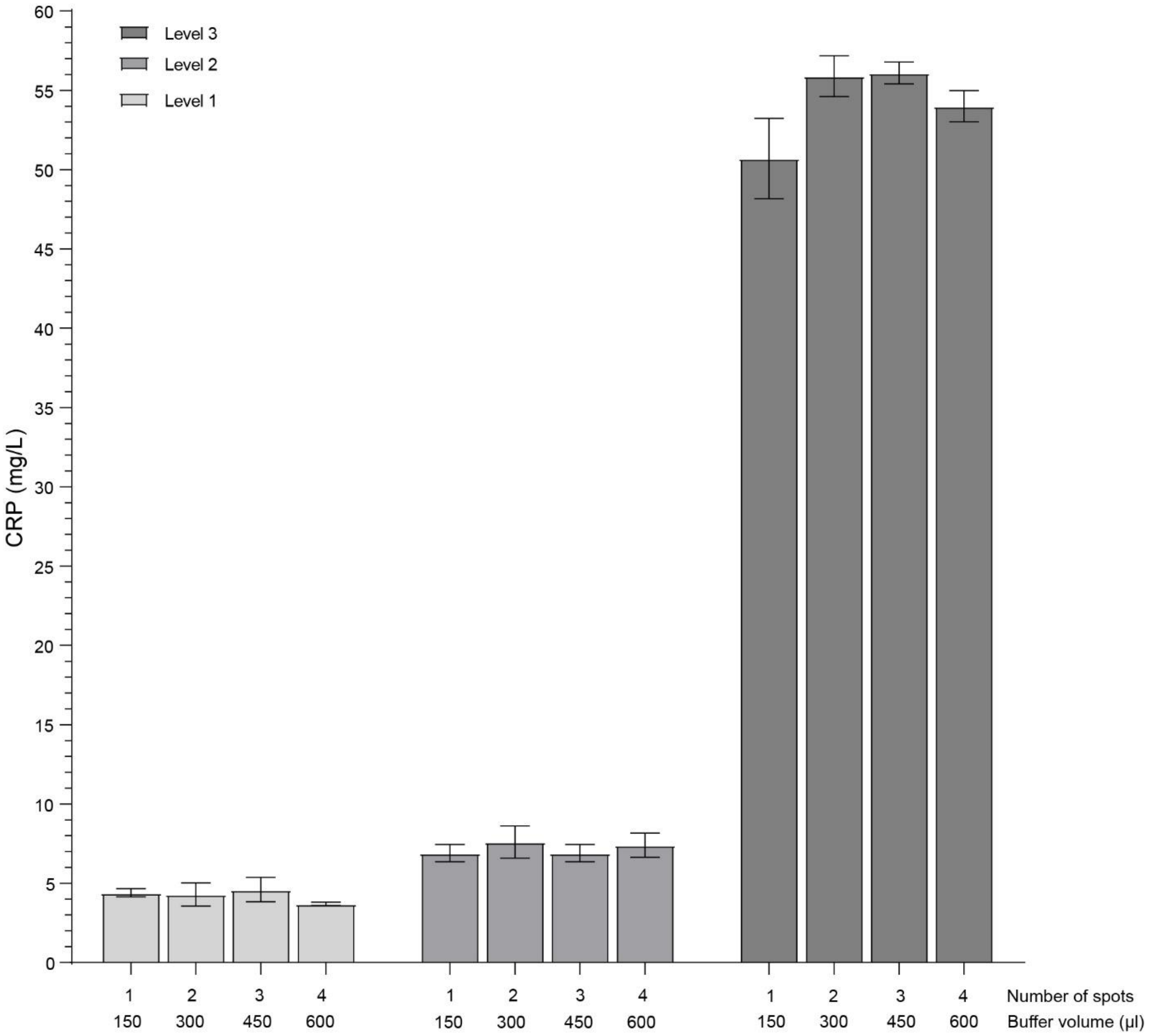

Supplementary figure 2 – Proportional correlation between the number of spots and extraction buffer volume.

Venous EDTA whole blood patient pools (n=3) with CRP target concentrations of 5.0 mg/L, 8.7 mg/L, and 54 mg/L were spotted. For extraction, either 1, 2, 3, or 4 spots per sample were used. For each spot, 150 µl PBS was added and extraction proceeded as normal. Samples were analyzed as described before

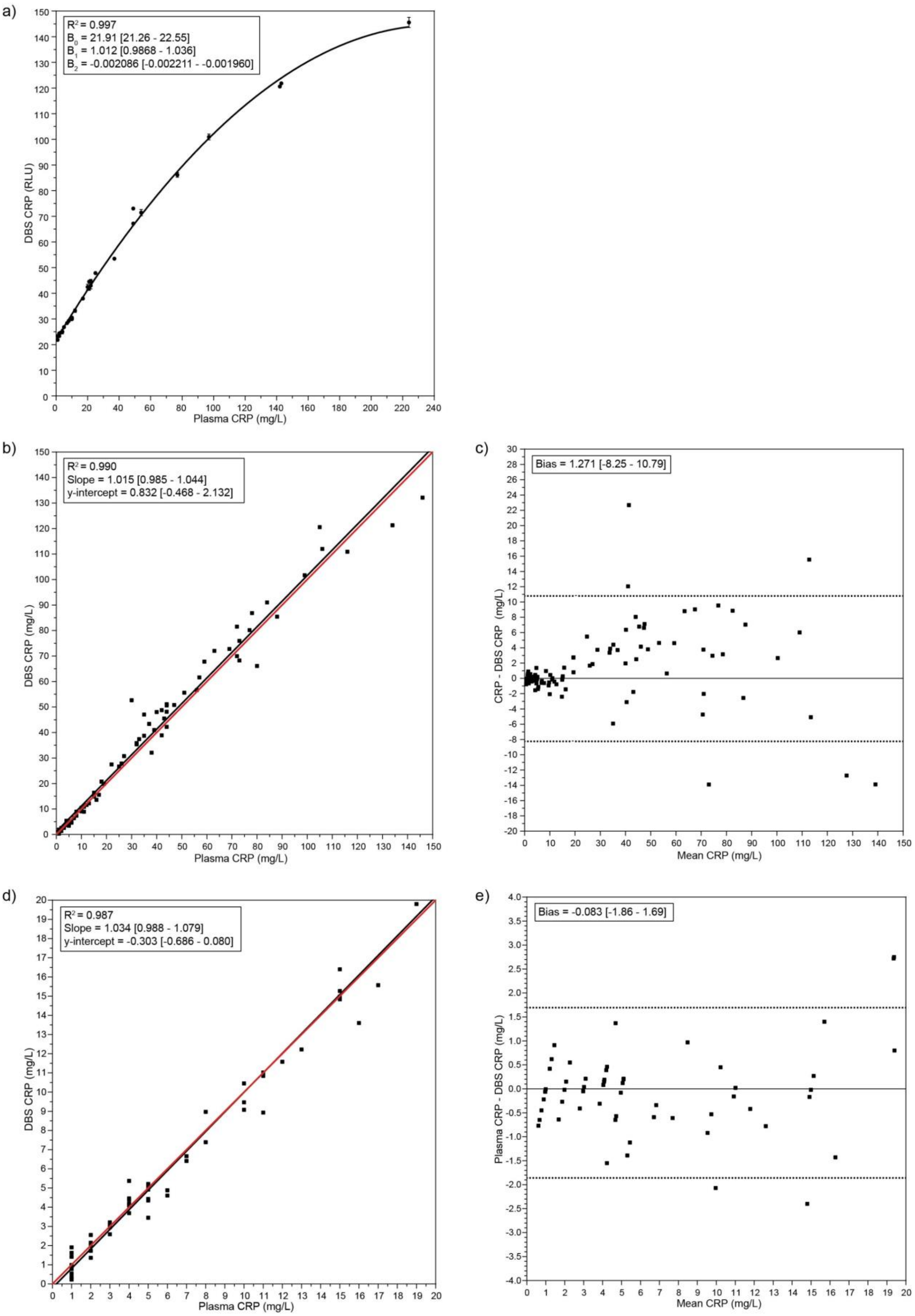

Supplementary figure 3 – CRP method comparison.

(A) Calibration line for DBS CRP (range 1 – 224 mg/L), calibration line was calculated with second-order polynomial regression analysis; n=28. (B) Method comparison for DBS CRP with the line of identity (red) and Deming linear regression (black); n=100 (CRP range: 1 – 146 mg/L, 50% male, average age = 57 years, age range = 1 – 87 years). (C) Absolute bias plot from method comparison of panel (B). (D) Method comparison for DBS CRP in near-normal range of CRP with the line of identity (red) and Deming linear regression (black); n=57. (E) Absolute bias plot from method comparison of panel (D). All 95% confidence intervals are shown in between brackets.
